# Short-term recurrent coronary artery thrombosis with acute myocardial infarction in a patient with aplastic anemia–paroxysmal nocturnal hemoglobinuria syndrome: a case report

**DOI:** 10.3389/fcvm.2025.1532842

**Published:** 2025-03-14

**Authors:** Xue-Guo Fu, Yan-Hua Guo, Shi-Chao Wang, Wen-Quan Zhang

**Affiliations:** Department of Cardiology, Qilu Hospital of Shandong University Dezhou Hospital, Dezhou City, Shandong, China

**Keywords:** aplastic anemia, paroxysmal nocturnal hemoglobinuria, acute myocardial infarction, cardiac troponin, myocardial injury, coronary thrombosis

## Abstract

**Background:**

Acute myocardial infarction commonly occurs in patients with coronary artery disease, but rarely, it can develop under a hypercoagulable state. Aplastic anemia can be accompanied by paroxysmal nocturnal hemoglobinuria clones or transform into paroxysmal nocturnal hemoglobinuria with a significantly elevated prothrombotic state. These thrombotic complications predominantly arise in veins rather than in arteries. Coronary artery thrombosis in these patients, especially with short-term recurrent arterial thrombosis after initial successful treatment, is exceedingly rare.

**Case presentation:**

A 39-year-old man with a history of aplastic anemia with paroxysmal nocturnal hemoglobinuria clones for 8 years presented with chest pain, and was diagnosed with acute inferior wall myocardial infarction on November 21, 2022. Despite standardized coronary intervention and anticoagulant/antiplatelet therapy, the patient reported intermittent chest discomfort with persistently elevated cardiac troponin and d-dimer levels 20 days after initial treatment. Repeat coronary angiography confirmed recurrent thrombosis in the right coronary artery. He underwent repeated balloon dilation and thrombus aspiration with intensified anticoagulation, which alleviated his clinical symptoms and normalized his cardiac troponin and d-dimer levels. The patient was finally confirmed to have aplastic anemia–paroxysmal nocturnal hemoglobinuria syndrome.

**Conclusion:**

Patients with aplastic anemia–paroxysmal nocturnal hemoglobinuria syndrome can have thrombosis in arteries, such as coronary arteries, leading to acute myocardial infarction. Recurrent coronary artery thrombosis can occur after initial successful revascularization and anticoagulant/antiplatelet therapy. Close monitoring of clinical symptoms, repeated electrocardiogram and laboratory tests, coronary angiography, strengthened anticoagulation, and precautions for bleeding risks should be considered in patients with aplastic anemia–paroxysmal nocturnal hemoglobinuria syndrome.

## Introduction

Acute myocardial infarction (AMI) describes the complete or incomplete occlusion of coronary arteries, resulting in a sudden reduction or interruption of blood supply and causing irreversible acute myocardial ischemia and necrosis (1, 2). The most common cause is coronary artery disease with atherosclerotic changes, which is often observed in older adults with multiple medical issues, such as hypertension, diabetes, and hyperlipidemia. In addition, AMI occasionally occurs in patients with a hypercoagulable state, such as those with paroxysmal nocturnal hemoglobinuria (PNH).

PNH is an acquired benign clonal disorder of hematopoietic stem cells caused by somatic mutations, and it clinically manifests as chronic intermittent intravascular hemolysis, thrombosis, and varying degrees of bone marrow failure (3, 4). PNH can evolve from aplastic anemia (AA). It was reported that more than half of patients with AA could carry PNH clones (5). Because of their proliferative advantage, PNH clones can replace bone marrow hematopoiesis, leading to the transformation of AA into PNH (6). The risk of thrombosis significantly increases after AA transforms into PNH, with venous thrombosis being more common than arterial thrombosis. Thrombosis commonly involves abdominal and intracranial veins (7–12). However, AMI with thrombosis in the coronary arteries has been occasionally reported in patients with PNH. Most of these reports were from Western countries, with a few cases identified in Northeast Asia (8). In this study, we report a Chinese patient with a medical history of AA accompanied by PNH clones. He had chest pain, and he was diagnosed with acute ST-segment elevation myocardial infarction (STEMI) and short-term recurrent coronary arterial thrombosis. We share our treatment experience regarding this patient to remind our colleagues about the extremely rare but life-threatening condition of STEMI arising from recurrent coronary arterial thrombosis in patients with AA-PNH syndrome.

## Case presentation

A 39-year-old man presented to our hospital on November 21, 2022 with sudden-onset chest pain for 4 h. He reported a medical history of AA (accompanied by PNH clones) that was diagnosed at another hospital 8 years before presentation. His medications included danazol and methylprednisolone. Upon presentation, the vital signs were as follows: temperature, 36.2°C; pulse, 80 beats/min; respiratory rate, 20 breaths/min; and blood pressure, 146/85 mmHg. The patient was alert with a poor mental status, no cyanosis in the lips, bilateral coarse breath sounds without rales on lung auscultation, and regular heartbeats with no murmurs. Otherwise, his physical examination was unremarkable. Laboratory testing revealed a white blood cell count of 10.7 × 10^9^/L, neutrophil count of 7.9 × 10^9^/L, monocyte count of 0.6 × 10^9^/L, red blood cell count of 3.4 × 10^12^/L, hemoglobin level of 104.0 g/L, hematocrit level of 33.4%, platelet count of 148 × 10^9^/L, high-sensitivity cardiac troponin level of 25,606.0 ng/L, and d-dimer level of 1,442 ng/ml (Table 1). The electrocardiogram (ECG) revealed sinus rhythm with ST-segment elevation in leads II, III, and aVF, suggesting acute inferior myocardial infarction. The patient was admitted into the hospital with diagnoses of acute inferior myocardial infarction and AA (accompanied by PNH clones).

**Table 1:** Dynamic changes in peripheral blood high-sensitivity cardiac troponin I and d-dimer levels in this patient.

Tests	Day 1	Day 5	Day 15	Day 21	Day 27 (hospital discharge)	Two weeks after discharge	One month after discharge	Four months after discharge
Troponin I	25,606.0	9,665.3	4,454.1	1,064.5	879.2	75.0	38.4	21.1
d-dimer	1,442	868.0	1,774.0	951.0	783.0	417.0	111.0	122

Reference ranges: cardiac troponin, 0–17.5 ng/L; d-dimer, 0–232 ng/ml.

**Figure 1:**
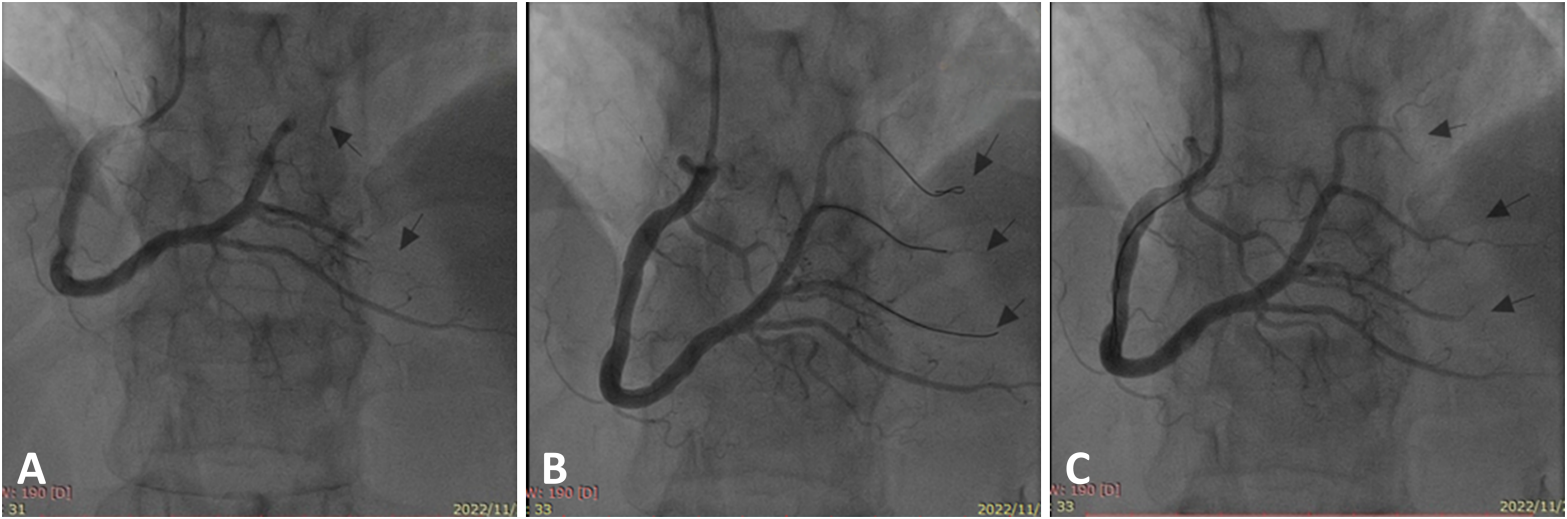
Right coronary artery under the initial coronary angiography. **(A)** Occlusion of the distal posterior left ventricular branch and its first branch (arrows). **(B)** The occluded site was opened by a guidewire (arrows). **(C)** After balloon dilation and thrombosis aspiration, the final angiography revealed smooth blood flow in the distal branches of the right coronary artery with no significant stenosis (arrows).

Emergency coronary angiography was performed, revealing a right-dominant coronary artery system with normal openings of the left and right coronary arteries. The left main stem displayed no significant stenosis. The left anterior descending and circumflex branches featured normal openings and courses without obvious stenosis. The right coronary artery was enlarged with a normal opening and course. The posterior left ventricular branch (PLB) of the right coronary artery exhibited distal occlusion, and the first branch of the PLB displayed distal occlusion with visible thrombus (Figure 1A). Considering the patient's history of AA and significant thrombotic burden, we decided to perform coronary angioplasty without stent placement. The guidewire smoothly passed through the occluded segment (Figure 1B). Using a compliant balloon, the stenotic segment was dilated. The thrombus was aspirated. Thrombolysis in Myocardial Infarction (TIMI) 3 flow was achieved in all branches of the right coronary artery and PLB, with no significant local stenosis observed (Figure 1C). The ST segment in leads II, III, and aVF significantly decreased on cardiac monitoring. The patient's chest pain disappeared.

Postoperatively, the patient received antiplatelet and anticoagulant therapies, including oral aspirin 100 mg once daily, clopidogrel 75 mg once daily, and intravenous tirofiban at a rate of 0.15 μg/kg/min for 24 h. Subcutaneous injections of low-molecular-weight heparin sodium 0.4 ml (units) were given twice daily for 1 week. In the following 2 weeks, the patient occasionally reported mild chest discomfort. Although the repeat ECG examination did not reveal any significant dynamic changes, the patient's serum cardiac troponin I and d-dimer levels remained high after 20 days (Table 1).

**Figure 2:**
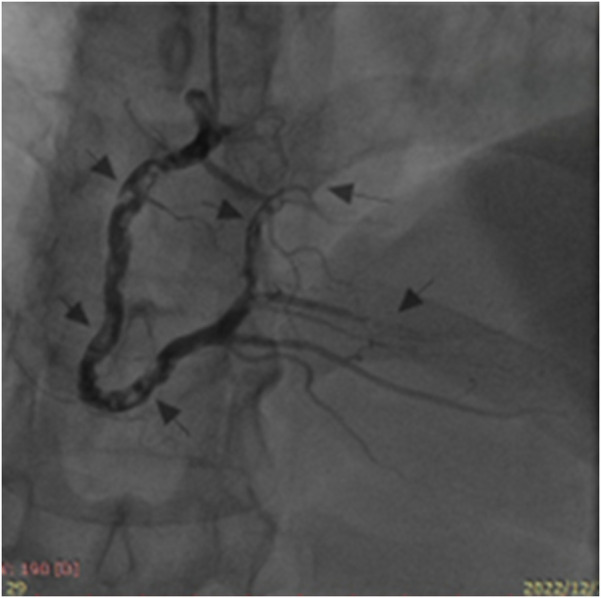
Second coronary angiography revealing diffuse thrombosis throughout the entire right coronary artery and occlusion of its distal posterior left ventricular branch and first branch (arrows).

We repeated coronary angiography on December 13, 2022, and no significant abnormalities were observed in the left coronary artery. However, diffuse thrombosis was present throughout the right coronary artery, in addition to occlusion of the distal PLB and its first branch (Figure 2). TIMI 3 flow was achieved after an intracoronary arterial injection of tirofiban (10 μg/kg). Postoperatively, tirofiban was continuously infused at a rate of 0.15 μg/kg/min for 24 h. The patient was subsequently prescribed long-term oral clopidogrel 75 mg and rivaroxaban 15 mg once daily. Repeated examinations revealed a significant decrease in the cardiac troponin I level, which gradually returned to normal (Table 1). He was discharged and followed up in the clinic.

In the clinic visits, the patient denied any chest pain or discomfort. Repeat testing revealed normal serum cardiac troponin and d-dimer levels. He also underwent follow-up at the hematology clinic, and he finally received a diagnosis of AA-PNH syndrome.

## Discussion

AMI commonly occurs in patients with underlying cardiovascular illnesses, such as coronary artery disease with hypertension and diabetes. Rarely, AMI can arise in patients with PNH. In this study, we reported a patient with a medical history of AA accompanied by PNH. He experienced short-term recurrent thrombosis in the coronary arteries, leading to sustained myocardial injury and persistent troponin elevation, which were successfully treated with balloon dilation, thrombus aspiration, and anticoagulant and antiplatelet treatment. We have reported this case to remind our colleagues to pay special attention to patients with underlying AA-PNH syndrome because their hypercoagulable state could occasionally cause serious and recurrent arterial thrombosis, leading to life-threatening illnesses.

AA and PNH are both rare hematologic disorders involving bone marrow and blood cells (3, 4, 13). They are distinct clinical diagnoses, but they share interconnected pathophysiological mechanisms. It was estimated that 20%–40% of patients with AA might have detectable PNH clones. When patients with AA have clinical features of PNH, AA-PNH syndrome is considered. The coexistence of AA and PNH can lead to a complex clinical course and prognosis, including a high risk of thrombosis. Prompt recognition of the hypercoagulable state is vital for preventing and diagnosing life-threatening thrombotic complications. Thrombosis in patients with AA-PNH predominantly arises in the hepatic, abdominal, or cerebral veins, but arterial thrombosis with severe morbidity and mortality rarely occurs. Short-term recurrent coronary artery thrombosis with myocardial infarction is exceedingly rare. A previous case report described a 33-year-old Chinese PNH female with two episodes of AMI. However, the authors failed to provide adequate evidence (such as coronary angiography) to support that two episodes of AMI were separate events (14). Our patient, who was previously diagnosed with AA and concurrent PNH clones, likely had coexistent AA and PNH, leading to a high risk of thrombosis. Under direct visualization, the thrombus in the right coronary artery was successfully removed. Routine antiplatelet and anticoagulant therapies were administrated postoperatively. Usually, cardiac troponin levels normalize within 2 weeks after AMI treatment. However, our patient had persistently elevated troponin and d-dimer levels after 20 days of treatment. Cardiac troponin is the standard marker for myocardial injury and/or necrosis. Its concentration can serve as a quantitative marker of myocardial cell damage to assess prognosis (2, 15). d-dimer levels can reflect the presence of thrombosis and fibrinolysis. Dynamic monitoring of d-dimer levels is valuable for the diagnosis, treatment, and prognosis prediction of thrombotic diseases, including acute cerebral infarction, AMI, deep vein thrombosis, and pulmonary embolism (16–18). A higher d-dimer level is associated with more severe coronary artery occlusion and a larger infarct size (19). We therefore performed repeat coronary angiography, which confirmed the presence of short-term recurrent coronary arterial thrombosis. After confirming that the coronary thrombus was the cause of the persistent elevation of troponin and D-dimer levels, we adjusted the treatment strategy, shifting the focus from antiplatelet to anticoagulation therapy. The patient has since been consistently taking the oral anticoagulant rivaroxaban. During follow-up, the patient has not experienced chest tightness or pain, and his cardiac troponin levels have remained normal. Further hematological evaluations ultimately led to a diagnosis of AA-PNH syndrome.

Our recommendation is that the possibility of arterial thrombosis with concerning clinical presentations should be eliminated in patients with a medical history of AA accompanied by PNH clones or AA-PNH syndrome. Those with chest pain should undergo appropriate diagnostic tests, including ECG and troponin level measurement. Emergency coronary angiography should be performed in patients with confirmed AMI. Balloon dilation and thrombus aspiration with follow-up enhanced anticoagulant therapy and routine antiplatelet treatment should be applied. Considering significant hypercoagulable state in AA-PNH patients, in-stent thrombosis could happen after coronary stent placement (20). Intensified anticoagulation, rather than stent placement, might be recommended in these patients. Postoperatively, close monitoring of troponin and d-dimer levels, and dynamic changes in ECG findings are crucial to detect recurrent thrombosis. Coronary angiography should be performed if there are persistent clinical symptoms of chest discomfort, abnormal ECG findings, or elevated troponin and d-dimer levels. Meanwhile, patients with AA-PNH syndrome can have an increased risk of bleeding disorders because of thrombocytopenia from AA and platelet dysfunction from PNH. They should be closely monitored for any sign of bleeding during anticoagulant and antiplatelet treatments. It was reported that thrombosis in PNH patients could be resistant to the anticoagulant treatment, The anti-C5 humanized monoclonal antibody, such as ravulizumab, could be applied to prevent recurrent thrombosis (21). In our patient here, intensified anti-platelet and anticoagulation treatments, including tirofiban, clopidogrel, and rivaroxaban, also achieved successful outcomes.

The initial coronary angiography in this patient revealed mild vascular stenosis with a heavy thrombotic burden at the distal end, indicating that the primary cause of vascular occlusion was thrombosis, with coronary artery disease being a secondary factor. Despite adequate anticoagulant and antiplatelet treatment following the diagnosis of AMI, the patient experienced recurrent thrombosis in the right coronary artery. Interestingly, two subsequent angiographies of the left coronary artery did not reveal thrombosis, suggesting that right coronary artery disease could exacerbate the risk of thrombosis in patients with AA-PNH syndrome.

## Conclusions

AMI can occur in patients with AA-PNH syndrome. Despite initial successful revascularization of the coronary artery, thrombosis can return with recurrent myocardial infarction. Close monitoring of clinical symptoms, repeated ECG and laboratory testing, necessary coronary angiography, strengthened anticoagulation, and precautions concerning bleeding risks should be considered in patients with overlapping hematological and cardiovascular disorders.

## Data Availability

The original contributions presented in the study are included in the article/Supplementary Material, further inquiries can be directed to the corresponding author.
